# Global Research Trends on Smart Homes for Older Adults: Bibliometric and Scientometric Analyses

**DOI:** 10.3390/ijerph192214821

**Published:** 2022-11-10

**Authors:** Yi-Kyung Hong, Ze-Yu Wang, Ji Young Cho

**Affiliations:** Department of Housing & Interior Design (AgeTech-Service Convergence Major), Kyung Hee University, Seoul 02447, Korea

**Keywords:** bibliometric analysis, scientometric analysis, smart home, older adults, VOSviewer

## Abstract

A growing aging population across the world signifies the importance of smart homes equipped with appropriate technology for the safety and health of older adults. Well-designed smart homes can increase the desire of older adults’ aging-in-place and bring economic benefits to the country by reducing budgets for care providers. To obtain a structural overview and provide significant insights into the characteristics of smart homes for older adults, this study conducted bibliometric and scientometric analyses. We used the Web of Science Core Collection database, searching for keywords “smart home*”, “home automation”, or “domotics” with terms related to older adults, resulting in a total of 1408 documents. VOSviewer software was used to map and visualize the documents. The results showed that research on smart homes for older adults began appearing from 1997 and increased steadily, peaking from 2015. The main research areas were technical engineering fields, such as computer science and engineering, telecommunications with minimal research in humanities, social sciences, and design, indicating the necessity to expand research toward a human-centered perspective, age-friendly technology, and convergence study.

## 1. Introduction

The global population aged over 65 totaled 771 million people in 2022 and is expected to reach 994 million in 2030, and 1600 million in 2050. The proportion of people aged 65 or over accounted for approximately 10 percent of the global population in 2022 and is expected to increase to nearly 16 percent in 2050 [1]. Older adults tend to spend a longer duration of time at home, with the tendency increasing in the wake of the COVID-19 pandemic. Staying most of their time at home results in older adults’ “disconnection with external assistance services” [2]; and “smart home technology is gaining more importance among this population” [2]. In addition, a decrease in the number of care providers resulting from low fertility facilitates the growth of smart home technology and industry that can contribute toward saving for care, promoting independent living of older adults, and Aging-In-Place (AIP). Smart homes, which provide safety, health, and convenience relevant to everyday life, have gained increasing attention from the industry and research sectors. Therefore, it is worthwhile to conduct research on smart homes for older adults systematically and comprehensively to identify the current status, provide future direction, and gain significant insights from them.

A smart home refers to “a residence equipped with smart technologies aimed at providing tailored services for users” [3]. The market for smart homes has been growing and is expected to increase from 22.3 trillion won in 2021 to 27.5 trillion won in 2025 in Korea [4]. Meanwhile, the worldwide smart home market is expected to grow to 173 billion US dollars in 2025 [5]. It can provide “residents with security, convenience, comfort, energy efficiency and entertainment and enhancing their lifestyle and domestic life” [6]. The expansion of Internet of Things (IoT) technology has accelerated the development of smart homes and use of diverse technology and services conveniently [7]. Smart home technology can aid residents with diverse characteristics, especially older adults’ AIP. It also contributes to “independent living among the elderlies, people with dementia or intellectual disabilities” [8].

Researchers have examined Smart Homes for Older Adults (SHOA) with diverse topics. For example, some researchers investigated the specific effects and potential of smart home technology for older adults. To illustrate, Fleury et al. (2010) studied Activities of Daily Living (ADLs) using data from diverse sensors in healthy smart homes [9]. Portet et al. (2013) investigated voice interface systems based on voice commands and found that they are potential solutions facilitating the daily lives of the elderly and the frail [10]. Blackman et al. (2016) studied whether Ambient Assisted Living (AAL) technology supports older adults’ independence and the quality of life and found that it helps uphold their health and well-being [11]. Wang et al. (2022) studied smartphone sensors with location monitoring systems that can be used in multi-class abnormal behavior recognition for older adults’ health and care [12].

In addition to technology-based research, there are studies on usability and perception of older adults toward smart home technology. For example, Demiris et al. (2004) investigated older adults’ perception and expectation for smart home technology for quality of life [13]. Additionally, Demiris et al. (2008) studied the necessity and preference for smart home technology by older residents [14]. Peek et al. (2014) examined a systematic literature review on factors influencing technology acceptance for AIP [15], and Meulendijk et al. (2011) proposed a planning principle for smart homes considering older adults’ motivation, preference, and experience for ambient intelligent domotics [16]. Liu et al. (2016) conducted a systematic literature review on the technology readiness level of older adults for supporting AIP and found that the level remains low [17]. Yu et al. (2020) analyzed the residentially-based lifestyles of older adults in Korea and revealed that there are significant differences in actual needs of smart home functions by groups of different lifestyles [2].

Meanwhile, the development of information technology and big data analysis has paved the way for the exploration and systematic categorization of bibliometric data; and there have been several attempts to systematically analyze smart home research. To illustrate, Li et al. (2022) analyzed bibliometric data (n = 17,189) published in Scopus from 2000 to 2021 using scientometric analysis and suggested historical changes, emerging trends, and research clusters [6]. Choi et al. (2021), using Scopus database from 2015 to 2019 on smart homes and IoT, conducted a bibliometric study and reported key trends and knowledge mapping [7]. Li et al. (2019) conducted bibliometric and scientometric analysis on smart city research and discovered main topics and knowledge areas [18]. Although not on smart homes, Oladinrin et al. (2021) examined 1331 research articles on AIP in 1970–2021 from Web of Science (WoS) using the scientometric analysis method [19].

However, to the best of our knowledge, there is no research that systematically analyzed research trends on smart homes, particularly for or related to older adults; the current study is the first to do so. A systematic analysis of prior studies on a certain topic and discovery of the main research trends is meaningful given that it offers significant insights grounded on bibliometric data and proposes a comprehensive knowledge base and mapping. Therefore, the purpose of this research is to investigate international research trends on SHOA using bibliometric and scientometric analyses to examine research trends, notable publication venues and authors, and research clusters, and to propose emerging areas and potential research gaps. We conducted analysis research trends by published year, country, organization, publication sources, research area, co-authorship among countries and organizations, and keyword co-occurrence and document co-citation analysis; and through such a process, we proposed recent trends and emerging areas on SHOA. The results from the analysis will provide research direction and focus for relevant stakeholders, such as researchers, policy makers, educators, and industry.

## 2. Materials and Methods

### 2.1. Collection of Data

For analysis, the study used the WoS database developed by Clarivate Analytics in the United States, which is a web-based database allowing users to search academic materials published after 1900. It contains a comprehensive database of literature from influential papers and has the most reliable downloading capabilities [19]. In the current research, we used the Core Collection database in WoS with “All” editions in Core Collection, which includes databases published in SCI-Expanded, SSCI, A&HCI, CPCI-S, CPCI-SSH, and ESCI indexes. The research method was “Topic”, which includes the research title, abstract, author keywords, and Keywords Plus. Keywords Plus is automatically generated from cited titles in WoS [20].

For searching terms, the following three keywords were used to get comprehensive documents on the subject: “smart home”, “home automation”, and “domotics”. Smart home is a relatively new term as in the past “home automation” and “domotics” were used [6]. In addition, we used “old adults” related terms such as “old man” OR “the aged” OR “the elderly” OR “elderly” OR “senior*” OR “older people” OR “old people” OR “old person” OR “older person” OR “older adult*”, which indicated both singular and plural words for the same term. We searched data published in English by publication types such as Proceedings Papers, Articles, Review Articles, Early Access, Meeting Abstracts, and Data papers. The search was conducted on 15 June 2022, and a total of 1408 documents were acquired. They were downloaded in text file format and converted into VOSviewer for network analysis.

### 2.2. Method of Analysis

We conducted bibliometric and scientometric analyses and used VOSviewer software for network analysis. Table 1 shows the research procedure.

#### 2.2.1. Bibliometric and Scientometric Analyses

Bibliometrics and scientometrics are novel methods for “measuring and analyzing scientific publications in a certain area” [21]. “Comprehensively understanding and the evaluation of the literature in a given field requires a more scientific bibliometric” [18]. Bibliometric analysis is one of the “innovative techniques for providing the current trends and improvements of specific knowledge domains” [7]. The term Bibliometrics was first introduced by librarian Pritchard in 1969, as “the application of mathematical and statistical methods to books and other media of communication. It included relationship among number of papers growth of literature and patterns of library database usage” [22]. It has been used to quantitatively analyze frontier trends in many research fields, including influential journals, countries, institutions, authors, much-studied fields, and ecological engineering, environmental sciences, financing and medicine, using mathematics and statistics [6,7,18,23,24].

Scientometric analysis is a method “to build bibliometric maps that how specific disciplines, scientific domains, or research field are conceptually, intellectually, and socially structured” [25]. It analyzes the quantitative aspects of the production, dissemination, and use of scientific information to better understand the mechanisms of scientific research [22]. When reviewing prior studies on bibliometric and scientometric analyses, the difference between the two is not clear. In this research based on Li et al.’s (2019) perspective, we consider general characteristics analysis such as frequency of publication by year, country, and author as bibliometric analysis, and network analysis among authors, organization, and making clusters of research using VOSviewer as scientometric, and we used both to achieve a comprehensive understanding of the topic [18].

#### 2.2.2. VOSviewer Software Program

For network analysis, we used VOSviewer (version 1.6.18) (Centre for Science and Technology Studies, Leiden University, The Netherlands), a software tool “for creating maps based on network data and for visualizing and exploring these maps” [26]. It is a free program developed for “constructing and viewing bibliometric maps” and “to create knowledge maps of the identified productive authors, core journals, contributing countries and organizations, influential documents, and co-occurring keywords” [19]. It has diverse functions such as co-authorship, co-occurrence, and co-citation analysis. It is suitable for big data-based research because it represents relationships of network data, knowledge structure, evolution, and collaboration [27].

## 3. Results

### 3.1. General Characteristics Analysis

#### 3.1.1. Yearly

**Figure 1:**
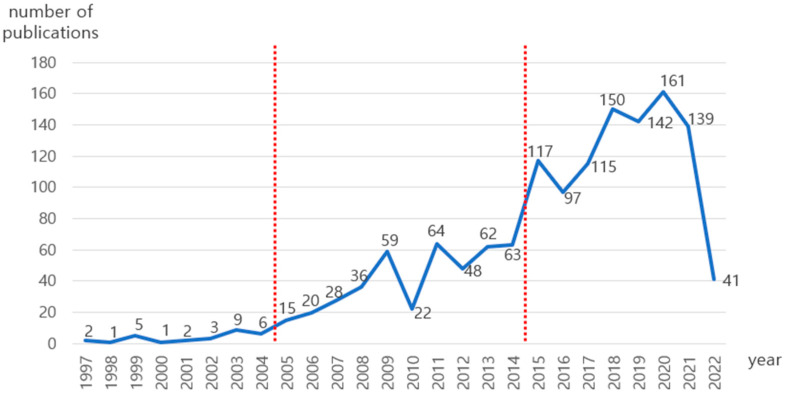
Number of publications by year.

The documents on SHOA studies were analyzed by year (Figure 1). The SHOA research can be seen in three stages. The first publication appeared in 1997. By 2004, fewer than 10 documents were published; this period is the beginning stage. The period from 2005 to 2014 shows a steady growth where the publications increased gradually up to 64. The rapid growth started from 2015, with over 100 publications per year.

The first period was the Beginning stage (1997–2004). A total of 29 publications (2.06%) belong to this period, with an annual average of 3.6. The term “domotics” was first used in the earliest study, “*A multimedia man-machine interface for the disabled and elderly*” [28] in 1997. Angelidis et al. (1997) noted that domotics is “the design of assistance machines that replace humans in performing household activities” [28]. Subsequently, Poulson (1997) in “*Using new technology to support the provision of care services*” [29] described a pilot work on supportable “home automation” technology for product and service development for the elderly and the disabled. In 1998, Nelisse and van Woerden (1998) used the term “smart-home” in “*ICAN—Integrated communication and control for all needs*” [30]. They found that new technologies such as mobile communication and smart home technology have a particularly important impact on the quality of life for the elderly and disabled.

The second period is the Steady growth stage (2005–2014). Overall, 417 publications (29.6%) belonged to this period, with an annual average of 41.7. Starting from 2005, studies on SHOA were promoted at various academic conferences, including “*International Conference on Smart Homes and Health Telematics*”, “*Sensor Networks and Information Processing Conference*”, and “*IASTED International Conference on Telehealth*”. Studies in the technical aspects were mainly introduced in this stage, such as smart home systems, sensors, ICT-based cloud computing, and embedded systems.

The increase in publications during this period seems to be related to the implementation plan for smart home-related technologies in various countries and institutions. The International Telecommunication Union released the World Telecommunication Development Report in 2003 in connection with Information and Communication Technologies [31]. In 2008, the National Intelligence Council identified IoT as one of the six innovative technologies that could affect national competitiveness by 2025 [32]. In 2009, the European Commission announced the “Internet of Things-An Action Plan for Europe“ [33]. Through the 12th Five-Year Plan on the IoT, China announced detailed plans for IoT innovation and major development directions and actively supported them at the government level [34]. In addition, in 2014, Apple launched the Apple HomeKit, a software framework that allows users to configure, communicate and control smart home appliances using Apple devices [35] and Amazon launched Alexa (Echo), a smart speaker with a voice-controlled intelligent personal assistant service [36]. The appearance of these products increased the interest in smart homes and provided an opportunity to directly experience the technologies of smart homes in real life.

The third period is the Rapid growth stage (2015–June 2022). A total of 962 publications (68.3%) belonged to this period, with an annual average of 120. In 2020, 160 documents were published, the largest number of publications.

These results indicate that SHOA have received great attention from the industry and academia in the last seven years. Li et al. (2022) found that the growth of smart homes is closely related to the development of IoT [6,37]. In addition, the increase in investment in smart technology and the strengthening of research funding can be considered as the causes for the increase in SHOA research at this time. The United States promoted investment in artificial intelligence and data science through the National Science Board’s Vision 2030 [38]. The National Institute of Standards Technology has promoted the *‘Global City Teams Challenge’*, which has evolved into a collaborative platform for city, community, industry, academia, and government stakeholders to jointly develop and distribute the latest technologies for smart cities and communities [39]. In Korea, smart homes have been added as the main task of the 4th Basic Science and Technology Plan (2018–2022), and government R&D investment in intelligent smart homes is increasing [4]. Japan’s Ministry of Education, Culture, Sports, Science and Technology introduced the ‘R&D project on ICT system architecture in the IoT era (2015–2020)’ for the purpose of establishing an international research environment [40]. In addition, in 2016, Google entered the smart home market by launching Google-Home, a smart speaker that allows users to control smart home appliances with voice commands or listen to music and play videos or photos. In particular, the influence of IoT is expected to spread to industries as well as daily life, as Gartner (2014) predicted that the production of IoT devices and services will increase significantly [41]. With the development of these smart home technology policies, industries, and technologies, SHOA research increased significantly during this period.

The number of publications in 2022 was limited as the data were collected only until June 2022. With rapid global aging and the development of technologies fostering through the Fourth Industrial Revolution, including artificial intelligence (AI), studies on SHOA continue to attract attention and are expected to grow in the future.

#### 3.1.2. Publication Sources

There were 200 publication sources in the field of SHOA. Table 2 shows the Top 10 most productive publication sources.

Among them, seven document types were Proceedings Papers, which were publications presented at the Conference, meaning that many impactful studies on SHOA are being presented and shared through the conference. The largest number of publications (a total of 117 documents) was from “Lecture Notes in Computer Science”, which is a distinguished conference proceeding series in all areas of computer science [42]. The other three are journals (“Sensors”, “IEEE Access”, and “Journal of Ambient Intelligence and Humanized Computing”).

When analyzing the C/P values to examine the number of citations and their influence, they were high in the order of “*Sensors*” (17.90), “*Journal of Ambient Intelligence and Humanized Computing*” (16.53), and “*IEEE Access*” (14.67). The higher the C/P value, the greater is the influence of the journal.

The main research areas of publication sources were identified through the JCR Category in WoS; and when no information is provided (Ranks 3, 5, 6, 8), we used information on the research area listed on each journal’s website. In the field of JCR Category research, more than one field is presented. The top 10 publications mainly covered computer science, engineering, and technology, including “*Computer Science*” (Artificial Intelligence, Information Systems), “*Engineering*” (Electrical & Electronic), and “*Telecommunications*”.

#### 3.1.3. Research Areas

The 1,408 documents covered 74 research areas. Table 3 shows the distribution of top 10 research areas on SHOA in the two-year period. The darker the shade of red, the greater the number of documents on SHOA that appeared in a particular year. The most relevant area of publications related to SHOA was “Computer Science” (783 publications), followed by “Engineering” (585 publications), “Telecommunications” (219 publications), “Medical Information” (125 publications), and “Health Care Science Services” (117 publications). It appears that the distribution of SHOA research is inclined toward “technology and engineering”. Instruments Instrumentation, Geriatrics Gerontology, and Automation Control Systems show a steady growth, while Chemistry shows recent growth. Rehabilitation shows a steady decrease considering the increasing number of total publications on SHOA.

Meanwhile, as a living venue for older adults, publications on residential environment and architecture/design were limited with only 28 publications. They are published in Environmental Science Ecology (21 publications), Architecture (5 publications), and Art (2 publications); many of them were conducted recently from 2020 to 2022. When looking at some of the studies in these areas, Yu et al. (2020) categorized the residential-based lifestyle of older adults and studied each group’s diverse needs for smart home functions [2]. Kwon et al. (2021) investigated the objective diagnosis method of dementia through automatic classification of early-stage dementia based on ADL data obtained in a smart home environment [43].

In summary, research on SHOA mainly focused on computer and technical engineering areas, while gerontology, medicine, and health areas are steadily gaining momentum. The residential and architectural environment areas accounted for a small proportion although they increased steadily after 2020.

### 3.2. Network Analysis

Network analysis using the VOSviewer program was performed to examine the research collaboration and network.

#### 3.2.1. Co-Authorship Analysis by Countries

Co-authorship network analysis between countries was conducted with those with a minimum of five publications included in the analysis. Among the 84 countries, only 50 met the criteria. Table 4 shows the top 10 most productive counties. The top three countries were the USA, China, and France. The USA is leading the SHOA field more than any other country. C/P indicates the number of citations per publication. The USA ranked first in both the number of publications and C/P, indicating its dynamic status in this field. This may be because of its “active aging society and high rates of funding for research“ [19].

While the C/P of the USA, French, and Canadian publications was higher than 20, China’s C/P was relatively lower although the number of publications is high. By continent, among the top 10 countries, 2 belong to North America, 4 to Europe, 3 to Asia and 1 Oseania.

The number of publications by country appear related to funding agencies because 3 of the top 10 funding agencies are in the USA (i.e., National Science Foundation, National Institutes of Health, and the United States Department of Health Human Services) and 2 are in Japan (i.e., Japan Society for the Promotion of Science, and the Ministry of Education, Culture, Sports, Science and Technology). Others are in the EU (European Commission), China (National Natural Science Foundation of China), Canada (Natural Sciences and Engineering Research Council of Canada), and Korea (National Research Foundation of Korea). These agencies belong to countries where active publications were produced. Research funding for the SHOA seemed to facilitate active research in the field in the country.

**Figure 2:**
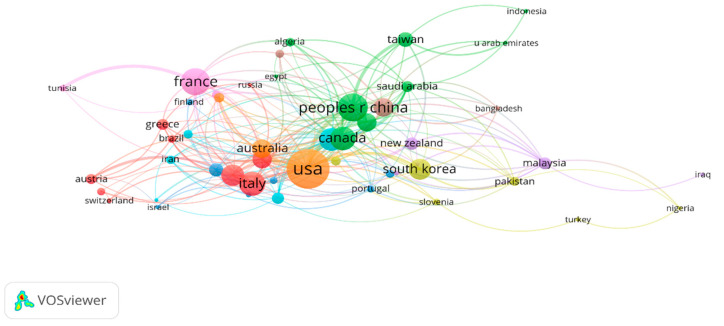
Network visualization by main countries.

Figure 2 shows the network among countries. As more papers are published in one country, the size of the node increases, and the country-to-country link line indicates the intimacy between the two countries: the thicker the line and having the same color, the closer the relationship between the countries. There are 9 clusters and 238 links in this network. The USA, China, Italy, and Canada are at the center and are closely connected to each other. The USA has a thick line connecting China, Canada, Korea, and Australia, indicating that these countries are closely connected. Close collaboration was identified through networks between the USA and China (total link strength = 11.33), the USA and Korea (total link strength = 7.50), and the USA and Canada (total link strength = 5.33). Countries with these networks ranked high in the number of publications as well.

#### 3.2.2. Co-Authorship Analysis by Organizations

Overall, 200 organizations conducted SHOA research, and the top 10 productive organizations are shown in Table 5. Among them, 79 organizations published a minimum of five publications. Massey University of New Zealand was the most productive organization, followed by the University of Washington and Washington State University in the USA, and CNRS (Centre National de la Recherche Scientifique) in France.

Organizations in North America, including the USA and Canada, occupy 6 of the top 10 positions, indicating that it is a major continent for SHOA. Other organizations are CNRS in France and Ulster University in the UK. Korea’s Kyung Hee University was the only one in Asia. In terms of the number of citations, CNRS was the highest, followed by the University of Toronto, Massey University, and Kyung Hee University.

**Figure 3:**
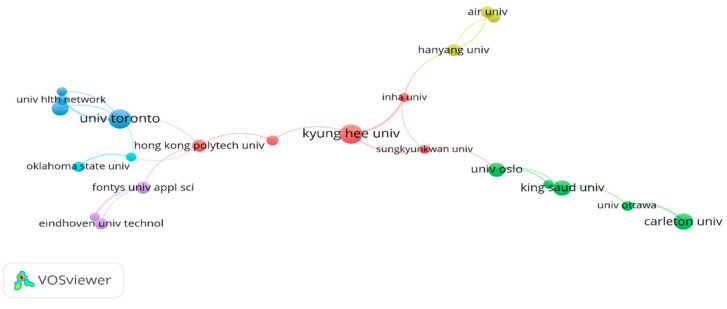
Network visualization by collaborative organizations.

Figure 3 shows a collaborative network among organizations that have published at least 5 documents, a total of 22 nodes, 6 clusters, and 24 links. The links connecting the two nodes indicate the strength of collaboration among organizations, and the thicker the line, the stronger the collaboration. Overall, there is not much collaboration across countries. The University of Toronto in Canada, which had a large node size, had a strong collaboration with the University Health Network in Canada (link strength = 5.00), while Kyung Hee University, Korea, showed a close collaboration with Inha University, Korea (link strength = 3.50). These strong collaborations occurred within the same countries as Oladinrin et al. (2021) mentioned, “Organizations from the United States, the United Kingdom, Europe, and Asia have succeeded in establishing collaborative relationships with each other” [19]. In conducting SHOA research, more collaboration within and across countries is necessary.

#### 3.2.3. Co-Authorship Analysis by Authors

In total, there were 200 authors in the 1,408 publications. To examine collaboration among authors, a co-authorship analysis was conducted using VOSviewer. With the minimum number of documents per author being 5, information of 79 authors who met the criteria was extracted. We excluded documents with more than 25 authors. Their countries, affiliations, number of publications, number of citations, and total link strength were analyzed for the top 10 productive authors (Table 6). Total link strength means “the total strength of the co-authorship links of a given researcher with other researchers” [26].

G. Demiris at the University of Pennsylvania in USA has published the largest number of publications among authors. He is the most influential author in SHOA with 12 link strengths, followed by S.C. Mukhopadhyay of Macquarie University, Australia, D.Cook of Washington State University in the USA, and B.Bouchard of Paris Dauphine University in France. Although M. Vacher at the Université Grenoble Alpes in France was ranked tenth, C/P value had the highest at 62.11.

**Figure 4:**
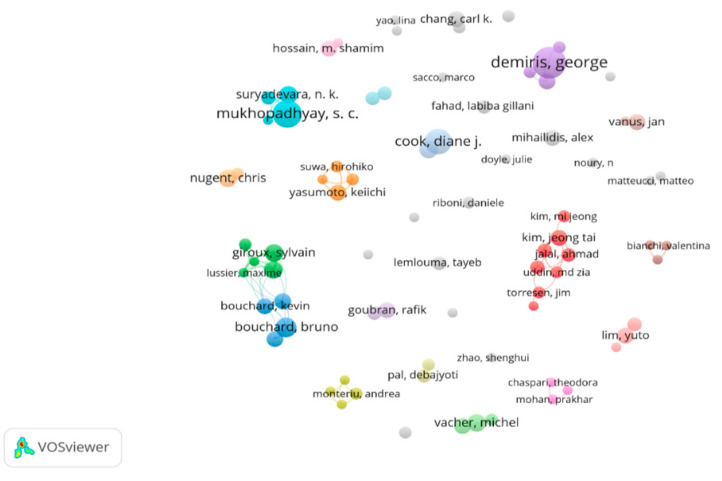
Network visualization by main authors.

Figure 4 is a visualization of networks by major authors, with 79 nodes and 35 clusters. Some of the top authors form closely connected groups, while others have relatively weak collaborative networks. Enhancing collaboration is important because “the more cooperation among contributors, the more they promote the development of the field” [18]. “Identifying and tracking network among authors is also crucial to broadening academic collaboration and communication by reducing isolation in research” [19].

### 3.3. Research Cluster Analysis

#### 3.3.1. Keyword Co-Occurrence Analysis

**Figure 5:**
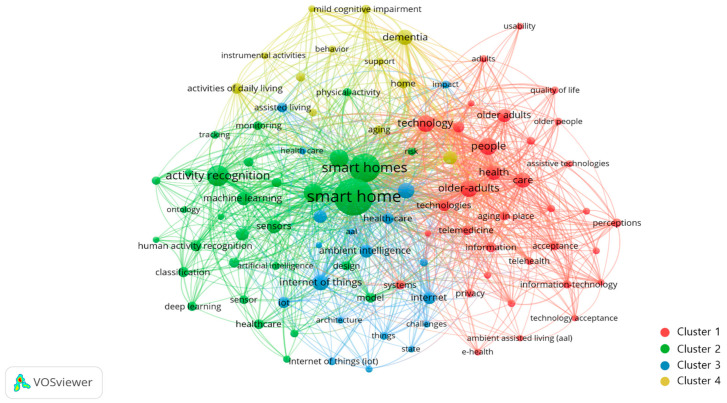
Co-occurrence network visualization.

Co-occurrence analysis was conducted to understand the distribution and relationship of the main topics of SHOA research because it can analyze “the internal relationship of an academic field” [18]. A map is created based on “keywords” of bibliographic data in co-occurrence analysis of the VOSviewer program. In this study, a total of 3842 keywords and 2422 links were generated. Through several optimization processes by varying the minimum number of keyword appearances, a clear keyword network visualization was created when setting the minimum occurrence number as 15. There were 95 keywords overall, and 4 clusters were created as follows: Cluster 1: older adults and smart home technology, Cluster 2: smart home, system, and activity, Cluster 3: IoT and environment for smart homes, and Cluster 4: aging and disease (Figure 5).

Cluster 1 is the most widely distributed keyword related to older adults and smart technology. Cluster 2 is in the center of the network around the “smart home” keyword. Keywords in Cluster 2 have the highest frequency of appearance and total link strength than those in other Clusters. Clusters 2 and 3 are adjacent and overlapping each other as keywords in the technical aspects of smart home, system, and IoT are closely related. Cluster 4 contains keywords about aging and disease and their number is limited.

We summarized the top 10 keywords in each cluster in Table 7. Cluster 1 has a total of 34 keywords such as people, older adults, technology, smart homes, health, and care. It proposes the need for a smart home to support the life and health of older adults. For example, Kuzman and Ayrilmis introduced independent smart homes as a clever co-living concept [44], and Street. et al. (2022) identified the acceptance and the effectiveness of smart technology of older adults (55+) in support of independent life [45].

Cluster 2 includes 30 keywords, such as smart home, smart homes, activity recognition, system, ambient assisted living, sensors, and machine learning. Through specific smart home technologies such as “sensors”and “deep learning”, it was attempted to understand the lives of humans (older adults).

“Smart home” and “smart homes” are considered two separate words in VOSviewer. Although they have the same meaning with one being singular and the other plural, the VOSviewer program does not treat them as the same. Relative to the human understanding of words, software algorithms cannot establish meaningful connections between words [46]. Some papers use “smart home” while some others use “smart homes”, and the software’s text mining algorithm divides these two words in the same cluster.

The smart home environment is an intelligent platform built over various conditions measuring sensors and communication technologies [47]. As a smart home technology component, a sensor is an input device that provides an output (signal) for a specific physical quantity (input) [48]. Recently, multiple sensor technologies with complex situational awareness are being developed that accept other types of information such as images and sounds through machine learning [49]. Machine learning is a type of artificial intelligence that allows software applications to become more accurate at predicting outcomes without being explicitly programmed to do so [50]. In this regard, Bouchabou et al. (2021) identified an algorithm for human activity recognition in the smart home through an ambient sensor [51]. Jo et al. (2021) evaluated the perception of the elderly about the integrated smart home system and pointed out that elderly participants perceived the necessity of using technology despite negative responses and emphasized the need to develop an elderly-friendly smart home sensor [52].

Cluster 3 has 19 keywords, including IoT, ambient intelligence, home automation, internet, and environment. The development of smart homes is one of the most important areas that have been significantly affected by IoT [7], on the basis of which users can interact with various devices and services in smart homes [53]. Debajyoti et al. (2021) explored the prohibitive factors for technology acceptance of IoT [54], while Pal et al. (2018) showed that IoT-based smart homes are an important solution for health care and services for the elderly from a user’s perspective. Effort expectancy, expert advice, perceived trust, and perceived cost represent the key influence of elderly peoples’ acceptance behavior [55]. They indicated that the emergence and applications of IoT have been consistently magnified in smart environments.

Cluster 4 includes 12 keywords, including dementia, activities of daily living, aging, assistive technology, and mild cognitive impairment. Globally, with the shift in aging and older adult care policies, interest in AIP is increasing with the aim of helping dementia patients live longer in their homes [56]. Moyle et al. (2021) suggested the role of smart home technology in supporting the health of older adults with dementia by mapping existing research on it [57]. Debes et al. (2016) pointed out that identifying deviations from previous patterns by monitoring the activities of the elderly in smart homes is important for early detection of crisis situations, enabling independent living in communities [58].

#### 3.3.2. Reference Co-Citation Analysis

Co-citation analysis evaluates the similarity of cited papers by tracing the linkage of the papers cited together in the source paper [59]. Moreover, it easily and conveniently identifies the core literature (knowledge base) in the relevant research field [18]. When two journals are referenced together, they are more closely related; influential research documents and author journals can be identified through co-citation analysis of sources [19].

**Figure 6:**
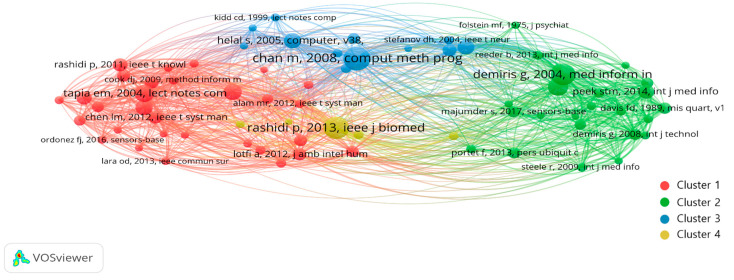
Co-citation network visualization of SHOA research.

In this research, co-citation network was visualized using the co-citation analysis of the VOSviewer program (see Figure 6). The minimum number of cited references was set to a default of 20, forming a total of 228 nodes and 4 clusters. Cluster 1 had 30 references and represented the largest node, Cluster 2 had 24, Cluster 3 had 12, and Cluster 4 had 5 references. On the map, the distance between two references indicates their relatedness in terms of co-citation links. In general, the closer the two references are placed, the stronger the relevance. “A co-citation link is a link between two items that are both cited by the same document” [26]. Table 8 shows reference with high co-citation in SHOA research.

Cluster 1: Elements of Smart Home Technology

There are 30 documents in Cluster 1. It deals with the subject of technological elements such as sensors and algorithms that construct smart homes along with the development of science and technology. The most cited reference in Cluster 1 is “*Activity Recognition in the Home Using Simple and Ubiquitous Sensors*” by Tapia et al. with 59 co-citations. The paper introduced a small and simple state change sensor, designed as a fast and ubiquitous device that can be used in many residential environments helping healthcare providers to recognize older adults’ activities [60]. Based on the research mentioned above, Suryadevara et al. (2013) designed and developed a new behavior detection process to be used in predicting the behavior and health of older adults in smart homes [61]. In “*CASAS: A Smart Home in a Box*”, Cook et al. (2013) emphasized the necessity to develop a lightweight design in smart homes and introduced CASAS “Smart Home in Box”, a customized lightweight home design that is easy to install [62]. Ni et al. (2015) analyzed activity characteristics and detection infrastructure for the independent life of the older adults in smart homes by reviewing several smart home projects including CASAS [63]. In “*SVM-Based Multimodal Classification of Activities of Daily Living in Health Smart Homes: Sensors, Algorithms, and First Experimental Results*”, Fleury et al. (2010) developed a method to automatically quantify ADL of older adults. For that purpose, a health smart home equipped with an infrared sensor, door contact, bathroom temperature and humidity sensor were used; the collected data was classified into ADL based on support vector machines (SVM)-that is a method to classify collecting data when the number of samples is limited [9]. Based on this, Donaj and Maučec (2019) presented a system for activity recognition and used a dataset from the CASAS to test and evaluate the proposed model. The core of these studies is to accurately grasp the daily living and activities of older adults from various sensor data [64].

Cluster 2: Factors for the Acceptance of Smart Technology by Older Adults

Cluster 2 included 24 studies. It focuses on factors influencing the perception of smart home technology and the acceptance of technology. The most frequently cited article is “*Older adults’ attempts towards and perceptions of ‘smart home’ technologies*”, published by Demiris et al. (2004), with 75 co-citations. The authors investigated the perceptions and expectations of the elderly for smart home technology installed and operated at home to improve their quality of life and monitor their health status [13].

Liu et al. (2016) conducted a systematic literature review on the level of technology readiness and AIP-enabled smart homes and home-based health monitoring technologies in their article, “*Smart homes and home health monitoring technologies for older people: A systemic review*”. They pointed out that the level of technical readiness of older adults for smart homes and home health monitoring technology was still low [17]. Peek et al. (2014) conducted a systematic literature review of factors influencing technology acceptance for AIP in “*Factors influencing acceptance of technology for aging in place: a systematic review*” and pointed out that acceptance of technology is influenced by several factors at the pre-use stage. They also suggested that further research is needed on interrelations among these factors as well as their relationship with existing technology acceptance models [15]. Based on this, Peek et al. (2016) explored the factors influencing the use of technology in older adults for AIP. In conclusion, older adults’ perceptions and use of technology are embedded in their personal, social, and physical contexts. The awareness of these psychological and contextual factors is needed to facilitate AIP via technology [65].

Cluster 3: The Future and Challenge of Smart Homes

There are 12 studies in Cluster 3. Most of the studies focus on future challenges on smart home development and discussions on priorities of smart home tasks. “*A review of smart homes-present state and future challenges*” by Chan et al. (2008) had the highest number of citations at 84. They emphasized that smart homes with built-in modern sensors can help people with physical disabilities, solve social isolation, and provide comfort, enjoyment, and happiness. This paper not only focuses on technologies such as wearable/implantable monitoring systems and assistive robotics but also leading smart home projects and future smart home challenges [66].

In “The Gator Tech Smart House: a programmable permanent space”, Helal et al. (2005) introduced the University of Florida’s The Gator Tech Smart house as a test platform that can aid older adults and disabled by implementing diverse systems and human-centric applications using pervasive computing technology [67]. “*Senior respondents’ paid needs of and preferences for ‘smart home’ sensor technologies*” is a study that examines the need and preference for specific smart home technologies (i.e., a bed sensor, gait monitor, stove sensor, motion sensor, and video sensor) of 14 elderly residents. Dermiris et al. (2008) reported that although the participants agreed to install sensors for nonobtrusive monitoring in their homes, older adults were concerned about privacy, so ethical and technical challenges need to be addressed [14].

The studies belonging to Cluster 3 are located close to Cluster 1, indicating that they are highly relevant (Figure 6). Both clusters have studies dealing with common smart home technologies, but Cluster 3 focuses on the future potential. Discussions were presented on the impact of smart homes on modern society and future prospects.

Cluster 4: Ambient Assisted Living and Healthcare

Seven studies belong to Cluster 4, which focus on healthcare and AAL or Ambient Assisted Living, a concept that combines ambient intelligence and assisted living to improve the independence and quality of life of older adults. “*A Survey on Ambient-Assisted Living Tools for Older Adults*” had the highest number of co-citations at 79. Total link strength was 348, which was the highest among all references. The authors summarized AAL technologies and tools that are helpful in independent living and proposed current and future challenges [68].

Another notable piece of research is “*Ambient Assisted Living Healthcare Frameworks, Platforms, Standards, and Quality Attributes*”, in which, Memon et al. (2014) emphasized that AAL is an emerging multidisciplinary field that utilizes information and communication technology of personal health care and telehealth systems to tackle the growing aging population. They provided a comprehensive review of the AAL field, focusing on the healthcare framework, platform, standards, and quality attributes [69].

In “*Ambient Assisted Living Technologies for Aging Well: A Scoping Review*”, Blackman et al. (2016) argued that AAL technology is an emerging field supporting the independence and quality of life of older adults and benefiting their health and well-being. They highlighted technologies available for people with normal to early stages of dementia and proposed theoretical considerations of the AAL geriatric perspective [11]. They also argued that multidisciplinary collaboration, such as social science and technical engineering, is necessary.

### 3.4. Emerging Trend of SHOA

**Figure 7:**
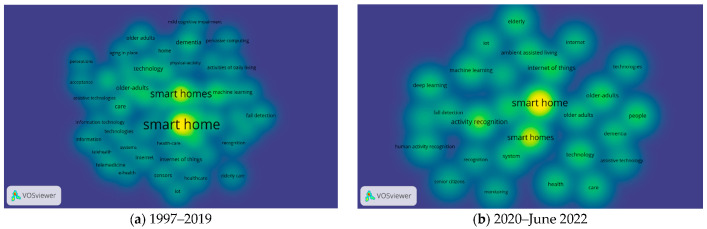
Distribution of SHOA research by keywords co-occurrence density map.

Emerging trend of SHOA research has been identified through analysis of recent trends of recent two- and half-year periods compared to those of the total period. For this purpose, density visualization of keywords co-occurrence in 1997–2019 as well as 2020–July 2022 was conducted. A total of 50 keywords in 1997–2019 and 26 keywords in 2020–July 2022 were obtained (Figure 7).

With the recent commercialization of 5G, a next-generation mobile communication service, and the emergence of services and hardware based on machine learning and artificial intelligence, the possibility of more systematic and efficient smart home implementation can be expected [70]. Since the development of each field—especially the recent explosion of artificial intelligence algorithms and computer performance—is an essential change, this section aims to examine differences from past research issues in the smart home field and provide basic data for future research.

In Density visualization, “The larger the number of items in the neighborhood of a point and the higher the weights of the neighboring items, the closer the color of the point is to yellow” [26]. When comparing keywords in recent years (2020–July 2022) with those in 1997–2009, terms seem to become more apart and more number of terms are visible in yellow, such as “internet of things”, “activity recognition, “technology”, “sensors”, “system”, “ambient assisted living”, and “health”; these indicate that these keywords have recently received considerable attention from researchers in the field of SHOA.

Table 9 shows emerging keywords in the recent period (2020–July 2022). For example, newly appearing keywords are visible, such as “deep learning”, “monitoring”, and “senior citizens”. In addition, keywords elevated more than 10 steps compared to the previous periods are “internet of things” and “sensors”. These newly emerging keywords show the tendency of evolving research focus.

An example of research on deep learning, one of the emerging terms, is Nugroho et al.’s study (2018) that introduced a smart home system detecting pain through facial expression analysis of older adults, developed based on deep learning technology [71]. IoT is another emerging term. A smart home is one of the most important areas affected by the development of “IoT“; this research area is called the smart home-Internet of Things [7].

Meanwhile, “dementia”, “care”, and “fall detection” declined recently although it is still important in SHOA. Examples on this topic are studies on technologies in detecting falls in a smart home environment [72,73], solutions to support the independent life of the elderly with dementia through smart homes equipped with simple sensor networks [74], and technology of smart home sensing and monitoring for dementia households [75]. Notably, ambient intelligence, home automation, telemedicine, independent living, activities of daily living, and classification disappeared from the top 25 keywords in the past three years.

## 4. Discussion

With the increase of the aging population and the ongoing crisis of COVID-19, smart home research focusing on “older adults” is rapidly growing. The overwhelming increase in the volume of literature related to SHOA has made it difficult for researchers and practitioners to have a comprehensive overview of SHOA. To obtain a structural overview and assist researchers in making insights into the characteristics of SHOA research, bibliometric and scientometric analyses were conducted in this paper. Through the results, it was confirmed that a smart home has tremendous potential in recent situations, such as an aging population and the pandemic, and has created a new research flow around the world [76].

### 4.1. Need for Diversity of Research Areas and Convergence Research

“Computer Science”, “Engineering”, and “Telecommunications” are the top three fields of SHOA research. They are mainly concentrated in the field of “technical engineering” and have seen an exponential growth in the last eight years (2015–June 2022). The above results reflect that “the existing knowledge structure of smart home research is more in favour of technical characteristics and advances” [6]. Conversely, research on architecture and environment for older adults is limited. Although the basis for smart homes is technology and computer science, research on the environment for older adults is essential as it is directly related to the quality of life. As Suh et al. (2015) mentioned, “studies on residential environment for the elderly have not actively been conducted so far” [77].

In addition, multidisciplinary convergence research across countries is needed in SHOA, including technology, older adults, and environment. Our finding shows limited collaboration between cross organizations and cross countries. Hong et al. (2019) identified the field of convergence between technologies of smart homes for older adults. It showed that the convergence of sensor technology and positioning technology was more widespread in the field of elderly smart homes [78]. Layton and Steel (2019) suggested that a smart home design should consider built environment, interfaces, and their interaction with residents (disabled and older adults) from an integrated perspective [79]. Suh et al. (2015) presented convergence research of cognitive information technologies in terms of cognitive sensor networks and architectural design for the elderly [77]. Appropriate funding will facilitate such convergence. For example, the Smart and Connected Communities program of NSF in the USA “supports integrative research that addresses fundamental technological and social science dimensions of smart and connected communities and pilots solutions together with communities” [80]. In that project, convergence research in technical computing, engineering, information and physical sciences, and social sciences has been emphasized to solve social problems.

### 4.2. Need for More Age-Friendly Technology and User (Human)-Centered Research

As the percentage of the aging population grows and life expectancy increases, research on older adults’ perspective of smart homes and user-centered studies are necessary. Li et al. (2021) pointed out that even publications on smart home research is increasing, “the existing research is still limited to the domains of technological advance, application prospects and constraints, and adoption intention of technologies” [76]. Azimi et al. (2017) also emphasized “there still exists a lack of user-centered study from an all-inclusive perspective for investigating the daily needs of senior adults” [81]. Carnemola (2018) noted that “research into AIP and home environments has focused on built environments and largely ignored the impact of technology in the lives of older people staying at home” [82]. The author conceptualized the interaction between smart home technology and the environment and verified the role of technology to support “AIP”. Suh et al. (2015) pointed out that various elderly care information systems are being developed to solve ultra-aging social problems, but these smart systems are not practical and useful for the elderly themselves [77].

Smart homes can be a great solution that supports independent and safe living and provides home health care. However, an important issue that should not be overlooked is the difficulties that older adults experience when they accept or interact with smart home technology. It is essential to focus on the “characteristics of smart home interface design and their impact on people of various ages” [83]. How people adopt smart home technology is “still an understudied area” [2].

In addition, as the evolution into a digital information society led by AI and IoT continues, older adults are facing problems such as social isolation and digital alienation. Studies based on age-friendly technologies need to be conducted when the use of interactive technology for connection with the outside world is essential for older adults, and studies related to person-environment-technology, technology acceptance, Geron-technology, home health, and wellness need to be addressed in the field of research. When searching keywords relevant to age-friendly technology in the co-occurrence analysis, no keyword was found with design exclusion, human-centered design, age-friendly, and user engagement. Very few studies have been conducted on user-centered design (7 times) and human–computer interaction (6 times), indicating that research in this field is extremely insufficient. Further research is needed to expand the SHOA to various research fields such as multidisciplinary convergence research on this subject [79] and explore the role of smart home technology in the future.

### 4.3. The Necessity for Research on Affordable Smart Homes with Health Services

The rise and decline of certain keywords indicate trends, but they also tell of shifts in funding to support such study areas. For example, the emerging keyword analysis showed an increase in machine learning, IOT, and sensors but a decrease in dementia, care, fall detection, and assistive technology; telemedicine disappeared from the top 25 keywords in the past three years. In addition, rehabilitation as a research area showed a decrease. This tendency is not attributable to a decrease in the number of patients diagnosed with dementia, but it may relate to limited services provided for patients at home. Studies have shown that still people feel unsafe and do not trust telemedicine, tele health, or home health monitoring systems [84]. In addition, the decrease in the number of publications about rehabilitation may mean that rehabilitation occurred mainly in healthcare settings, not in smart homes. Recently, telemedicine provided at home or the concept of the smart home as a health platform has attracted more attention. Still, concern about privacy and trust issues exists, so greater inclusion of care aspects in smart homes seems essential to reduce the burden for care providers and to increase older adults’ healthy living as well as AIP.

One important aspect for AIP is to make smart homes affordable and accessible for more people. To make a house a smart home, minimal infrastructure and the simple addition of devices and sensors are necessary [85]. Smart home technologies available only at newly constructed houses create little impact in the lives of ordinary older adults. Simple and easy methods of installation in existing structures will bring about more benefits to more people. Engaging the housing and technology sectors and integrating health delivery systems in the home will bring about real impact to the quality of life [86].

## 5. Conclusions

This study conducted bibliometric and scientometric analyses on SHOA research to better understand research publication trends, productive authors and organizations, co-authorship, co-citations, research cluster based on keywords, and emerging trends. Overall, 1408 documents were used in the analyses that was acquired from WoS Core Collection by searching the keywords of SHOA-relevant terms. Conclusions are as follows:

1. Globally, SHOA research is increasing by year with a rapid growth from 2015.
2. When analyzing by country, the USA, China, and Canada are the leading countries in SHOA. A strong network with the USA is visible with sound research funding. However, network and collaboration among different countries is not viable, which indicates the necessity for more collaborations across organizations and countries.
3. The main research clusters on SHOA are (a) older adults and smart home technology, (b) smart home, system, and activity, (c) IoT and environment for smart home, and (d) aging and disease.
4. The main clusters of the core literature (knowledge base) on SHOA are (a) elements of smart home technology, (b) factors for the acceptance of smart technology by older adults, (c) future and challenges of smart homes, and (d) ambient assisted living and healthcare.
5. Emerging keywords on SHOA research are deep learning, monitoring, IoT, sensors; activity recognition; and health-related keywords, such as healthcare and health show a steady growth.
6. The main research areas for publications were technical fields, such as computer science and engineering, with minimal research in the humanities, social sciences, and design, indicating the necessity to expand research to a diverse human-centered perspective.
7. The rise and the decline of certain keywords seem relevant to funding as well. Greater effort in diversifying funding sources and priorities seems necessary.
8. Considering the issue of digital segregation of older adults, research on age-friendly technology aspects is scarce. It is imperative to launch more efforts on research on age-friendly technology, such as a convergence study on diverse areas and extension of research topics, considering older adults’ psychological and physiological conditions.
9. More research on affordable and accessible smart homes with health services seems necessary.

This study has the following implications. First, it is, to the best of our knowledge, the first study that analyzed global research trends on SHOA with comprehensive and systematic method of bibliometric and scientometric analyses. The results from this research provide quantitative data on key knowledge basis that can be used to determine any gaps in research areas and collaboration and to plan for future research directions. Second, the results are useful for researchers, policy providers, public sectors regarding system, structure of knowledge base on SHOA, so that they can make evidence-based decisions on funding, policies, and investment priorities on research area. Third, the results will be a useful guideline in making global and national visions on smart home industry.

The research has the following limitations. First, as we used documents acquired from the WoS database published in English, we may have missed valuable documents published in non-English languages. Second, as the number of clusters is defined by the minimum number of co-occurred documents in keyword co-occurrence, research clusters proposed in this research are not absolute; instead, they can evolve as new publications appear. In this research, we did several optimized trials and set the minimum number of publications as 15 as it resulted in 4 clusters. Third, there might be a gap due to author name duplication and spellings in WoS data and the VOSviewer transfer process. The issue of treating singular and plural for a term with the same meaning as separate words in VOSviewer should be solved in the future.

Therefore, it seems essential to further develop the VOSviewer programs comprehensively and develop more diverse network analysis and visualization software to reduce any gap in data collection and analysis. It is the authors’ hope that the results from this research are helpful to the community of environmental research and public health, and development of agenda and policies on SHOA.

## Figures and Tables

**Table 1:** Research method flowchart.

Data Collection	Database	WoS Website Core Collection
	Search Criterion	“All” edition: SCI-Expanded, SSCI, A&HCI, CPCI-S, CPCI-SSH, ECI
		“Topics”
		languages: “English”
		Document types: Proceedings Papers, Articles, Review Articles, Early Access, Meeting Abstracts, Data papers
		Time span: –15 June 2022
	Keywords Input	Step 1: “smart home*” OR “home automation” OR “domotics”
		Step 2: “old man” OR “the aged” OR “the elderly” OR “elderly” OR “senior*” OR “older people” OR “old people” OR “old person” OR “older person” OR “older adult*”
Data Analysis	Analysis Tool	VOSviewer Software Program_1.6.18
	Method of Analysis	Bibliometric and Scientometric Analyses
		(1) General Characteristics Analysis: By Year, publication source, research area (2) Network Analysis: Co-authorship analysis> By country, organization, author (3) Cluster Analysis: Co-occurrence analysis: keyword Co-citation analysis: reference (4) Emerging Trend of SHOA research Keywords co-occurrence density visualization

**Table 2:** Top 10 most productive publication sources.

Rank	Publication Title	Number of Publication	Citation	C/P	IF	Core Collection Editions	JCR Category
1	Lecture Notes in Computer Science	117	709	6.06	0.97	CPCI-S	Artificial Intelligence
2	Sensors	71	1,271	17.90	3.847	SCI-Expanded, SSCI	Chemistry, Analytical Engineering Electrical & Electronic
3	Assistive Technology Research Series	40	64	1.6	-	CPCI-S	Engineering Medicine
4	IEEE Access	24	352	14.67	3.476	SCI-Expanded, SSCI	Computer Science, Information Systems Engineering, Electrical & Electronic Telecommunications
5	Studies in Health Technology and Informatics	22	127	5.77	0.78	CPCI-S	Engineering Biomedical Engineering Electrical & Electronic
6	IEEE Engineering in Medicine and Biology Society Conference Proceedings	20	285	14.25	1.12	CPCI-S	Biomedical Engineering Computer Vision & Pattern Recognition Health Informatics Signal Processing
7	Toward Useful Services for Elderly and People with Disabilities	19	138	7.26	0.302	CPCI-S	Computer Science, Artificial Intelligence
8	Advances in Intelligent Systems and Computing	16	52	3.25	0.61	CPCI-S	Computer Science Control & Systems Engineering
9	Journal of Ambient Intelligence and Humanized Computing	15	248	16.53	3.662	SCI-Expanded	Computer Science, Artificial Intelligence Computer Science, Information Systems Telecommunications
10	Lecture Notes in Artificial Intelligence	14	66	4.71	0.302	CPCI-S	Computer Science, Artificial Intelligence

Note: C/P = Citation/Number of Publication, JCR category = Journal Citation Reports (2021) Category, CPCI-S: Conference Proceedings Citation Index-Science; Data sources: WoS.

**Table 3:** Distribution of top 10 research areas on SHOA in two-year period.

Research Area	1997–1998	1999–2000	2001–2002	2003–2004	2005–2006	2007–2008	2009–2010	2011–2012	2013–2014	2015–2016	2017–2018	2019–2020	2021–2022	Total
Computer Science	2	1	4	14	15	36	50	62	67	136	160	154	82	783
Engineering	1	5	4	6	7	30	31	40	59	92	106	126	78	585
Telecommunications	0	0	1	2	1	11	16	12	17	31	46	52	30	219
Medical Informatics	0	0	0	2	5	12	4	8	12	24	22	23	13	125
Health Care Sciences Services	1	0	0	1	17	16	4	5	7	18	13	22	13	117
Instruments Instrumentation	0	0	2	1	1	1	1	9	11	9	17	36	26	114
Chemistry	0	0	0	0	0	0	0	0	8	5	16	30	24	83
Geriatrics Gerontology	0	0	0	0	2	5	3	5	4	13	15	14	9	70
Rehabilitation	2	4	1	7	11	6	3	7	2	2	8	7	0	60
Automation Control Systems	1	0	0	1	1	7	2	4	7	9	7	12	7	58

The red background indicates the density of publications.

**Table 4:** Top 10 most productive countries.

Rank	Country	Number of Publications	Citation	C/P	Total Link Strength
1	USA	209	5730	27.42	57
2	China	120	1362	11.35	53
3	France	114	2794	24.51	31
4	Italy	96	1195	12.45	30
5	Canada	93	1939	20.85	31
6	England	86	1703	19.80	41
7	Germany	79	852	10.78	23
8	South Korea	75	999	13.32	16
9	Australia	61	670	10.98	26
10	Japan	61	282	4.62	16

Note: C/P = Citation/Number of Publications.

**Table 5:** Top 10 most productive organizations.

Rank	Organization	Country	Number of Publication	Citation	C/P	Total Link Strength
1	Massey Univ	New Zealand	26	672	25.85	6
2	Univ Washington	USA	20	465	23.25	6
3	Washington State Univ	USA	20	305	15.2	4
4	CNRS	France	17	926	54.47	12
5	Univ Ulster	UK	17	281	16.53	0
6	Kyung Hee Univ	South Korea	15	368	24.53	6
7	Univ Toronto	Canada	15	739	49.27	8
8	Carleton Univ	Canada	12	189	15.75	4
9	Iowa State Univ	USA	12	178	14.83	0
10	Univ Sherbrooke	Canada	12	59	4.92	3

Note: C/P = Citation/Number of Publication.

**Table 6:** Top 10 most productive authors.

Rank	Author	Country	Affiliation	Number of Publication	Citation	C/P	Total Link Strength
1	Demiris, George	USA	Univ of Pennsylvania	26	672	25.85	6
2	Mukhopadhyay, S.C.	Australia	Macquarie Univ	20	465	23.25	6
3	Cook, D.J.	USA	Washington State Univ	20	305	15.2	4
4	Bouchard, Bruno	France	Univ Paris-Dauphine	17	926	54.47	12
5	Giroux, Sylvain	Canada	Univ of Sherbrooke	17	281	16.53	0
6	Nugent, Chris	North Ireland	Ulster Univ	15	368	24.53	6
7	Pigot, Helene	Canada	Univ of Sherbrooke	15	739	49.27	8
8	Schmitter-Edgecombe, Maureen	USA	Washington State Univ	12	189	15.75	4
9	Suryadevara, N.K.	India	Geethanjali College of engineering & technology	12	178	14.83	0
10	Vacher, Michel	France	Univ Grenoble Alpes	12	559	62.11	3

Note: C/P = Citation/Number of Publication.

**Table 7:** Cluster and top 10 keywords.

Cluster 1: Older Adults and Smart Home Technology			Cluster 2: Smart Home, System, and Activity			Cluster 3: IoT and Environment for Smart Homes			Cluster 4: Aging and Disease		
Keyword	Occurrence	Total Link Strength	Keyword	Occurrence	Total Link Strength	Keyword	Occurrence	Total Link Strength	Keyword	Occurrence	Total Link Strength
people	94	453	smart home	404	1018	internet of things	77	293	dementia	72	319
technology	87	408	smart homes	245	848	elderly	76	275	assistive technology	58	226
older-adults	86	425	activity recognition	136	387	ambient intelligence	52	187	activities of daily living	39	120
care	71	349	system	96	366	home automation	52	110	home	34	145
health	68	311	ambient assisted living	95	339	internet	48	221	aging	27	104
older adults	58	211	sensors	58	244	iot	42	128	mild cognitive impairment	26	125
technologies	44	226	machine learning	55	176	health-care	38	218	wireless sensor networks	26	60
independent living	37	175	fall detection	48	139	assisted living	32	92	pervasive computing	21	45
information	35	155	human activity recognition	39	111	internet of things (iot)	22	67	support	19	105
telemedicine	33	120	classification	36	120	environment	21	74	behavior	18	83

**Table 8:** Reference with high co-citation in SHOA research.

Cluster	Title	Author/s	Year	Publication	Co-Citation	Total Link Strength
1	Activity Recognition in the Home Using Simple and Ubiquitous Sensors	Tapia et al.	2004	Pervasive Computing	59	306
	CASAS: A Smart Home in a Box	Cook et al.	2013	Computer	54	265
	SVM-Based Multimodal Classification of Activities of Daily Living in Health Smart Homes: Sensors, Algorithms, and First Experimental Results	Fleury et al.	2010	IEEE Transactions on Information Technology in Biomedicine	51	231
2	Older adults’ attitudes towards and perceptions of ‘smart home’ technologies: a pilot study	Demiris et al.	2004	Medical Informatics and the Internet in Medicine	75	270
	Smart homes and home health monitoring technologies for older adults: A systematic review	Liu et al.	2016	International Journal of Medical Informatics	60	229
	Factors influencing acceptance of technology for aging in place: a systematic review	Peek et al.	2014	International Journal of Medical Informatics	46	260
3	A review of smart homes- present state and future challenges	Chan et al.	2008	Computer Methods and Programs in Biomedicine	84	322
	The Gator Tech Smart House: a programmable pervasive space	Helal et al.	2005	Computer	51	216
	Senior residents’ perceived need of and preferences for “smart home “sensor technologies	Demiris et al.	2008	International Journal of Technology Assessment in Health Care	49	193
4	A Survey on Ambient-Assisted Living Tools for Older Adults	Rashidi et al.	2013	IEEE Journal of Biomedical and Health Informatics	79	348
	Ambient Assisted Living Healthcare Frameworks, Platforms, Standards, and Quality Attributes	Memon et al.	2014	Sensors	27	70
	Ambient Assisted Living Technologies for Aging Well: A Scoping Review	Blackman et al.	2016	Journal of Intelligent Systems	26	92

**Table 9:** Keyword occurrences and emerging keywords in SHOA (1997–2019 and 2020–July 2022).

	1997–2019		2020–July 2022		
Keyword		Occurrences	Keyword		Occurrences
smart home		286	smart home	-	123
smart homes		172	smart homes	-	74
activity recognition		83	activity recognition	-	54
system		67	internet of things	●	38
ambient assisted living		66	older-adults	△	37
people		63	technology	△	34
technology		53	people	▽	33
older-adults		50	sensors	●	32
dementia		49	ambient assisted living	▽	29
care		48	system	▽	29
elderly		47	elderly	-	29
assistive technology		43	health	△	27
health		43	machine learning	▲	26
ambient intelligence	√	42	older adults	△	26
home automation	√	42	dementia	▼	24
internet of things		39	care	▼	24
fall detection		32	deep learning	★	23
older adults		32	internet	△	20
machine learning		29	iot	▲	20
internet		28	health-care	▲	18
sensors		27	technologies	△	18
telemedicine	√	27	fall detection	▼	17
independent living	√	26	human activity recognition	▲	17
technologies		26	monitoring	★	15
activities of daily living	√	25	assistive technology	▼	15
classification	√	24	senior citizens	★	15

Note: ▲: 5 steps or more, △: 1 to 4 steps up, -: no change, ▽: 1 to 4 steps down, ▼: drop more than 5 steps, ●: emerging more than 10 steps, ★: new keywords, √: disappear in top 25 keywords in 2020–July 2022.
